# Rapamycin Is Neuroprotective in a Rat Chronic Hypertensive Glaucoma Model

**DOI:** 10.1371/journal.pone.0099719

**Published:** 2014-06-12

**Authors:** Wenru Su, Zuohong Li, Yu Jia, Yehong Zhuo

**Affiliations:** State Key Laboratory of Ophthalmology, Zhongshan Ophthalmic Center, Sun Yat-sen University, Guangzhou, China; University of Regensburg, Germany

## Abstract

Glaucoma is a leading cause of irreversible blindness. Injury of retinal ganglion cells (RGCs) accounts for visual impairment of glaucoma. Here, we report rapamycin protects RGCs from death in experimental glaucoma model and the underlying mechanisms. Our results showed that treatment with rapamycin dramatically promote RGCs survival in a rat chronic ocular hypertension model. This protective action appears to be attributable to inhibition of neurotoxic mediators release and/or direct suppression of RGC apoptosis. In support of this mechanism, *in* *vitro*, rapamycin significantly inhibits the production of NO, TNF-α in BV2 microglials by modulating NF-κB signaling. In experimental animals, treatment with rapamycin also dramatically inhibited the activation of microglials. In primary RGCs, rapamycin was capable of direct suppression the apoptosis of primary RGCs induced by glutamate. Mechanistically, rapamycin-mediated suppression of RGCs apoptosis is by sparing phosphorylation of Akt at a site critical for maintenance of its survival-promoting activity in cell and animal model. These results demonstrate that rapamycin is neuroprotective in experimental glaucoma, possibly via decreasing neurotoxic releasing and suppressing directly apoptosis of RGCs.

## Introduction

Glaucoma is a leading cause of severe irreversible visual impairment and blindness. A common feature of glaucoma is elevated intraocular pressure (IOP) that results in the progressive loss of retinal ganglion cells (RGCs). Current treatments that lower IOP through surgeries and medicines cannot completely prevent the progressive degeneration of RGCs. This reflects an incomplete understanding of the pathways that lead to RGC loss in glaucoma. Thus, the development of new strategies that prevent RGC death and slow disease progression has become the primary goal of glaucoma therapy [1], [2].

Rapamycin, a lipophilic macrolide antibiotic, was developed as an antifungal agent but has versatile nonantibiotic properties [3], [4]. Recently, several studies have shown that rapamycin has neuroprotective effects in neurological diseases, including Parkinson’s disease [5], Alzheimer disease [6], nerve injury [7] and tuberous sclerosis [8]. However, the underlying mechanism of rapamycin-mediated neuroprotection remains elusive. The role rapamycin plays in glaucoma is also unclear. Thus, in this study we explored the role of rapamycin in neuroprotection in experimental glaucoma and the potential cellular and molecular mechanisms. We found that rapamycin can protect RGCs from death in a rat chronic ocular hypertension model (COH) of glaucoma. Mechanistically, we found that rapamycin is capable of inhibiting neurotoxic mediator release in microglia by modulating nuclear factor-kappa B (NF-κB) signaling *in* *vitro* and in the COH. Our results also showed that rapamycin directly suppressed apoptosis of RGCs by maintaining phosphorylation of Akt at Thr308. To the best of our knowledge, these findings provide the compelling evidence that rapamycin is neuroprotective in experimental glaucoma.

## Materials and Methods

### Ethics Statement

This study strictly adhered to the ARVO Statement for the Use of Animals in Ophthalmic and Vision Research and was approved and monitored by the Institutional Animal Care and Use Committee of Zhongshan Ophthalmic Center (Permit Number: SYXK (YUE) 2012–0024). Female Sprague-Dawley rats (8–10 weeks, 200–250 g) were obtained from the Guangzhou Animal Testing Center. They were maintained under a 12 h light/dark cycle in a temperature- and humidity-controlled room and given *ad libitum* access to food and water. Additional enrichment and welfare were provided; for example, Animal health was monitored daily by the animal care staff and veterinary personnel. All surgery was performed under sodium pentobarbital anesthesia, and animals were kept warm during and after operation. All efforts were made to minimize suffering.

### Animals and Experimental Glaucoma Model

A total of 70 Sprague-Dawley rats were studied in adherence with the ARRIVE guidelines. Rats were randomly assigned to study groups or control groups by an investigator not performing surgical procedures or data analysis. Induction of ocular hypertension by episcleral vein cauterization was performed on the right eyes as previously reported [9], [10]. In brief, rats were anesthetized with an intraperitoneal injection of sodium pentobarbital (50 mg/kg body weight). Three episcleral vessels were cauterized in the right eye with a 30-min cautery tip after anesthesia. The left eye was used as a normal IOP control after sham surgery (conjunctival incisions with no cauterization) in each animal. Using a Tonopen XL tonometer under anesthesia, IOP was measured immediately after cauterization of vortex veins and every week after cauterization surgery until the endpoint of each experiment. Five consecutive stable readings were obtained from each eye.

### Antibodies and Reagents

Rapamycin, glutamate, lipopolysaccharides (LPS) and an Akt inhibitor (Akt-1/2) were purchased from Sigma (St. Louis, MO). Antibodies included anti-Caspase-3, anti-Thy-1, (abcam, Hong Kong, China), anti-Akt (Thr308 and Ser473), anti-NF-κB p65, anti-I kappa B-alpha (IκB-α), anti-induced nitric oxide synthase (iNOS), anti-β-actin (Cell Signaling Technology, Inc., Danvers, MA) and anti-ionized calcium-binding adapter molecule (Iba) 1 (woka, Chuo-Ku, Osaka Japan).

### Rapamycin Preparation and Treatments

Rapamycin was dissolved in DMSO (25 mg/ml) and stored at −20°C. It was diluted immediately before final intraperitoneal injection with 0.5 ml aqueous solution containing 5% polyethylene glycol 400 and 5% Tween 80 [7]. Rats subjected to the COH were randomly divided into 2 groups of 8. COH rats in the rapamycin group received intraperitoneal injection of rapamycin (4 mg/kg) daily for 2 d before cauterization and every other day thereafter for 6 weeks. The control group received the drug vehicle.

### Fluorogold Retrograde Labeling of RGCs

The rats were anesthetized and mounted onto a stereotactic apparatus (Kopf Instruments, Tujunga, CA). The skull was exposed, and bilateral holes were drilled 6.0 mm from bregma and 1.0 mm from the midline. Using a Hamilton (Reno, NV) syringe, 3 µl of 4% Fluorogold (FG, hydroxystilbamidine; Invitrogen, Carlsbad, CA) in distilled water was injected into both superior colliculi at a depth of 5.0 mm from the brain surface. After the injection, the micro-syringe was kept in place for one minute to allow dispersion of FG into the superior colliculi. Finally, the skin over the wound was sutured with 5-0 silk sutures. In the glaucoma model, retrograde labeling was performed at day 35 after ocular hypertension (7 days before the experimental end point). This time afford excellent labeling efficacy and are practical and compatible with experimental procedures.

### RGC Survival Quantification

Quantification of labeled RGCs was performed as reported previously [10]–[13]. Seven days after retrograde labeling, the eyes were enucleated and fixed in 4% paraformaldehyde for 1 h. The retinas were flat-mounted on glass slides and dissected via four radial cuts to facilitate flattening of the retinas into a Maltese cross shape. Then, the retinas were observed under fluorescence microscopy (Carl Zeiss, Jena, Germany) with 12 pictures at 20 magnification. For each retina, there were 3 pictures from each quadrant at a distance of 1 mm, 2 mm, and 3 mm radially from the optic nerve (indicated as areas 1, 2, and 3). Microglia and macrophages that incorporated Fluorogold after phagocytosis of dying RGCs were excluded from our analysis according to their morphology, as reported [10]–[13].

Manual RGC counting was done by two independent persons. One person was the experimental performer, blinded to the drug treatment code, and the second person was unrelated to the experiment and was blinded to the entire protocol. Standardization of RGC counts and RGC loss in the test eyes (right eye, OD) were performed versus RGCs counted in contralateral normal IOP control eyes (left eye, OD, 100% RGC counts). Mean ± SEM was calculated for all percentages of RGC survival data, for each experimental group.

### Cell Culture

Primary RGCs were obtained from female Wistar rats according to a two-step immunopanning protocol described by Charanjit and Viswanathan [14], [15]. In brief, retinas were dissociated from 6-8-day-old Wistar rats to produce a suspension of single cells. The retinal suspension was incubated in OX-42-coated flasks to promote glial cell adherence. The non-adherent cells were placed in Thy-1-coated flasks. Finally, adherent cells were collected and incubated at 37°C in 5% CO_2_ with serum-free Neurobasal medium (Invitrogen, Carlsbad, CA) containing glutamine, B27 supplement, brain-derived neurotrophic factor, ciliary neurotrophic factor, gentamicin and forskolin.

Microglia BV2 cells (Shanghai Cell Bank of Academia Sinica, Shanghai, PRC) were cultured in high glucose (4.5 g/ml) DMEM medium (Gibco) supplemented with 10% fetal bovine serum and 1% penicillin/streptomycin at 37°C in a humidified incubator under 5% CO_2_.

### Assays for RGC Viability and Apoptosis

Cell viability and proliferation were measured using the VybrantH MTT Cell Proliferation Assay Kit (Invitrogen, Carlsbad, CA) according to the manufacturer’s protocol. Apoptosis of RGCs was determined by flow cytometry using a propidium iodide (PI)-PE and Annexin V-FITC detection kit according to the instructions provided by the manufacturer (Keygen Biotech, Shanghai, China). Flow cytometric analysis was performed following standard protocols.

### Western Blotting

The retina or cell homogenates (20–25 µg of total protein) were separated on a polyacrylamide-SDS gel and electroblotted onto a nitrocellulose membrane (Bio-Rad, Hercules, CA). After blocking with TBS/5% nonfat dry milk, the membrane was incubated with antibodies against rat NF-κB, IκB-α, caspase-3, iNOS and Akt followed by incubation with a horseradish peroxidase-conjugated secondary antibody. The signals were visualized by enhanced chemiluminescence detection (Pierce, Rockford, IL). The blots were also reprobed with a specific antibody against β-actin.

### Nitric Oxide (NO) Assay and ELISA

Supernatant NO levels were measured using the Griess reaction [16], [17]. In brief, 50 µl of the samples was mixed with 0.1% N-1-napthylethylenediamine dihydrochloride and 1% sulfanilamide at room temperature for 10 minutes. The absorbance at 550 nm was measured with a microplate reader.

Caspase-3 activity and nitrotyrosine content in the retinas were detected using the Caspase-3/CPP32 colorimetric assay kit (Biovision, Milpitas, CA) and OxiSelectTM Nitrotyrosine ELISA Kit (Cell Biolabs, Inc. San Diego, CA), respectively. Concentrations of TNF-α in retina lysates or in supernatants of cultured cells were detected by ELISA (eBioscience, San Diego, CA).

### Real-time PCR

Total RNA from cell lysates or rat retina lysates was extracted with the RNeasy Mini Kit (Qiagen, Valencia, CA), and cDNA was generated using an Omniscript RT kit (Qiagen). TNF-α and Iba1 mRNA expression was quantified using ABsolute SYBR Green ROX mix (Thermo, Waltham, MA). The samples were run in triplicate, and the relative expression of TNF-α and Iba1 was determined by normalizing the expression of each target to β-actin using the 2^−ΔΔCt^ method.

### Statistical Analysis

All data are expressed as the mean ± SEM from at least 3 independent experiments, and statistical analyses were performed with Student t test using SPSS software (16.0; SPSS, Chicago, Ill). Differences were considered statistically significant at a *P* value of less than 0.05.

## Results

### The Neuroprotective Effects of Rapamycin in the COH

**Figure 1:**
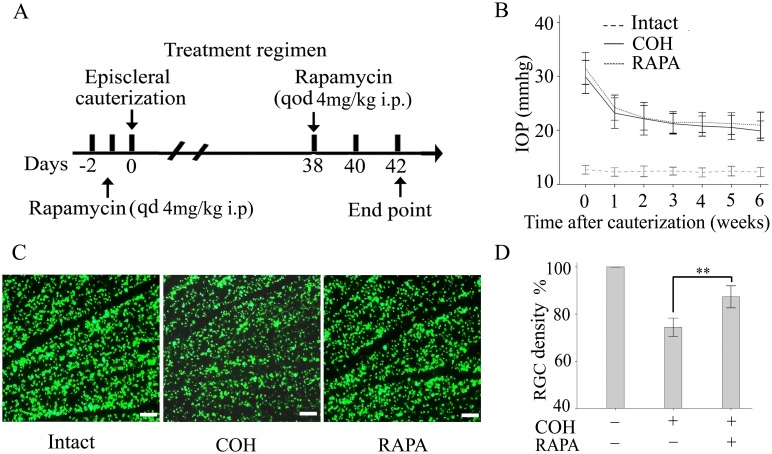
The neuroprotective effects of rapamycin in the COH. (A): Experimental protocols showing the treatment regimen of rats subjected to the COH receiving intraperitoneal injection of rapamycin (4 mg/kg) daily for 2 d before cauterization and then every other day for 6 weeks. (B): IOP was measured at the indicated time points after challenge in different groups. (C): Representative images of fluorogold-labeled RGCs of retinas in a normal and a COH rat. (D): Quantification of fluorogold labeling of RGCs. Each group includes 8 rats, and experiments were repeated once. The data are either representative or the mean ± SEM of 2 separate experiments. **p<0.01. Abbreviations: COH, chronic ocular hypertension model; RAPA, rapamycin; IOP, intraocular pressure; RGC, retinal ganglion cell.

To evaluate the role of rapamycin in glaucoma neuroprotection, we adapted a rat chronic ocular hypertension model that is a well-established animal model of human glaucoma. Rapamycin was administered intraperitoneally daily for 2 d before cauterization and then every other day for 6 weeks (Fig. 1A). In the COH, ∼1.7-fold elevation in IOP was maintained until the end point of each experiment. Rapamycin treatment did not alter the IOP compared with the control treatment (Figure 1B). Untreated COH eyes experienced a significant RGC loss compared with normal control eyes at the 42-day endpoint. Treatment with rapamycin resulted in 95% of labeled RGCs at the 42-day endpoint, which is significantly different from the control group that had 74% of labeled RGCs (Fig. 1C, 1D).

### Rapamycin Treatment Inhibited Apoptosis Signaling and Neurotoxic Mediator Release in COH Retinas

**Figure 2:**
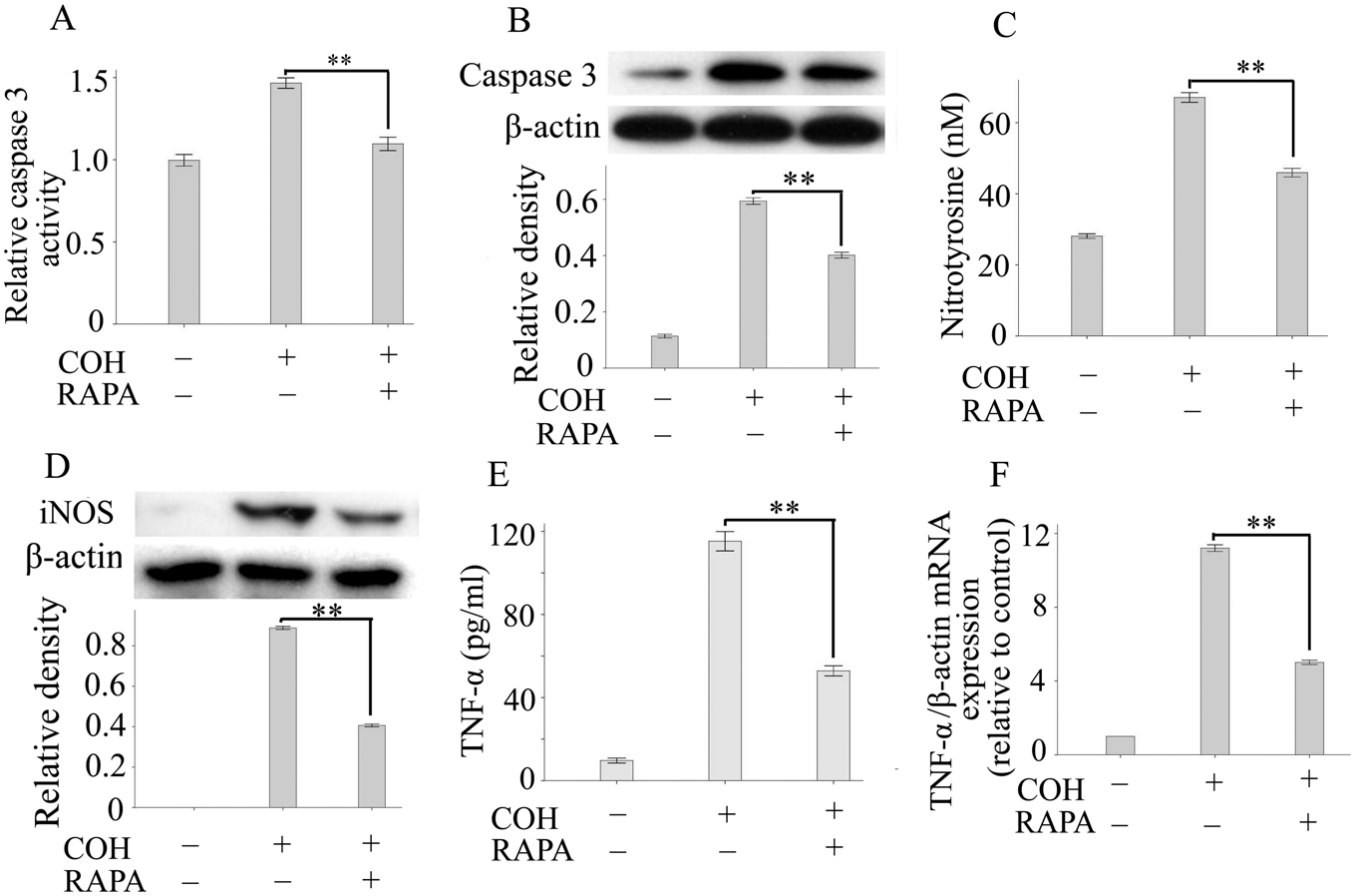
Rapamycin treatment inhibited apoptosis signaling and neurotoxic mediator release in COH retinas. Caspase-3 activity (A) and the expression of active caspase-3 (B) in retinas were detected by ELISA and western blotting, respectively. (C): The nitrotyrosine content in retinas was determined using the OxiSelectTM Nitrotyrosine ELISA Kit. (D): The level of iNOS in retinas was measured by western blotting. TNF-α protein (E) and mRNA expression (F) was determined based on real-time PCR and ELISA. Each group includes 8 rats, and experiments were repeated once. Values are presented as the mean ± SEM of 3 replicates. **P<0.01. Abbreviations: COH, chronic ocular hypertension model; RAPA, rapamycin; iNOS, induced nitric oxide synthase; TNF-α, tumor necrosis factor-alpha.

Previous studies have shown that caspase signaling-dependent apoptosis plays important roles in the primary and secondary waves of RGC apoptosis in glaucoma [2]. Thus, to investigate whether rapamycin improves RGC survival by decreasing apoptosis, we analyzed the level of active caspase-3 in retinas. As shown in Figure 2A, treatment with rapamycin significantly reduced the level of active caspase-3 in the COH retinas compared with untreated groups. The results of western blot analysis further confirmed rapamycin-mediated inhibitory effects on caspase-3 activation (Fig. 2B). Neurotoxic mediators, including reactive oxygen species (ROS), NO and TNF-α, have been implicated in secondary waves of RGC apoptosis in glaucoma. Thus, we next assayed the level of nitrotyrosine (derivative of ROS), iNOS and TNF-α in COH retinas. Our results showed a significant increase in the production of nitrotyrosine and TNF-α in tissue lysates of glaucoma retinas compared with those of normal controls, whereas treatment with rapamycin significantly decreased the local production of these neurotoxic mediators (Fig. 2C, 2E). The results from western blot and real-time PCR analysis also showed that treatment with rapamycin significantly reduced the expression of iNOS protein and TNF-α expression in retinas compared with the untreated group (Fig. 2D, 2F). These findings suggest that rapamycin is capable of improving the survival of RGCs, possibly by inhibiting the production of neurotoxic mediators and/or directly suppressing their apoptosis.

### Rapamycin Inhibits Neurotoxic Mediator Release in Microglia by Modulating NF-κb Signaling

**Figure 3:**
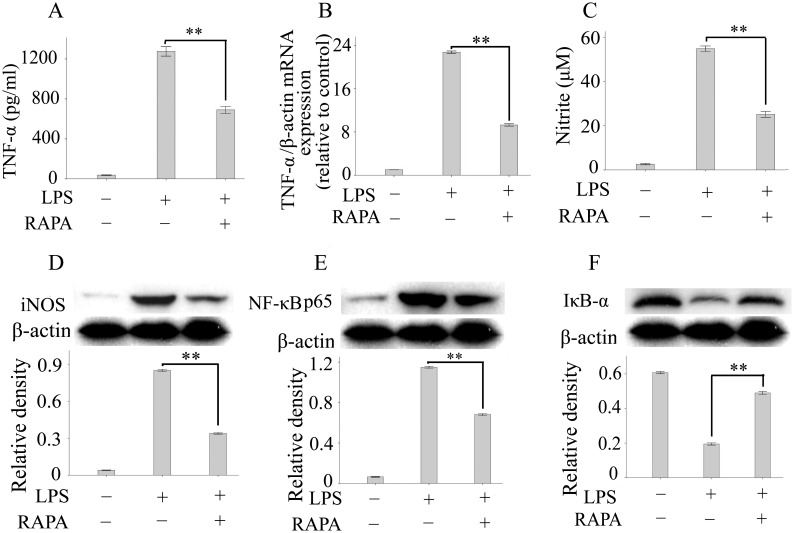
Rapamycin inhibited neurotoxic mediator release in microglia by modulating NF-κB signaling. BV2 microglia were plated in six-well plates for 24 hours and subsequently stimulated with LPS for another 16 hours in the presence or absence of rapamycin. The TNF-α level (A) in the supernatants and the expression of TNF-α mRNA (B) in microglia were determined by ELISA and real-time PCR, respectively. (C): The nitrite content in the supernatants was measured by the Griess reaction. The expression of iNOS (D), NF-κB (E) and IκB-α (F) in microglia was determined based on western blotting. Values are presented as the mean ± SEM of 3 replicates, and experiments were repeated at least 2 times with similar results. **p<0.01. Abbreviations: LPS, lipopolysaccharides; RAPA, rapamycin; TNF-α, tumor necrosis factor-alpha; iNOS, induced nitric oxide synthase; NF-κB, nuclear factor-kappa B; IκB-α, I kappa B-alpha.

Microglial activation and microglia-derived neurotoxic mediators, including TNF-α, NO and ROS, have been shown to play detrimental roles in neuron death and glaucoma. More importantly, previous studies have shown that inhibition of microglial activation is neuroprotective in experimental glaucoma [18]–[20]. Thus, we next studied whether rapamycin can inhibit the activation of microglia. BV2 cells, an established mouse microglia line, were plated in six-well plates for 24 hours and subsequently stimulated with LPS for another 16 hours in the presence or absence of rapamycin (100 nM). The production of TNF-α and NO in the supernatants of BV2 cells was measured by ELISA and the Griess reaction. Our results showed that rapamycin treatment led to significant inhibition of LPS-stimulated TNF-α and NO release by BV2 cells (Fig. 3A, 3C). Western blotting and real-time PCR results also showed that rapamycin significantly reduced the expression of TNF-α mRNA and iNOS protein in LPS-stimulated BV2 cells (Fig. 3B, 3D). NF-κB is a heterodimeric transcription factor that plays a key role in inflammatory mediator release and microglial activation [21]–[24]. Thus, we investigated whether rapamycin can modulate NF-κB signaling in microglia. Although stimulation of BV2 cells with LPS resulted in increased NF-κBp65 protein production, treatment with rapamycin significantly inhibited NF-κBp65 expression in BV2 cells (Fig. 3E). Most agents, including LPS, activate NF-κB through phosphorylation or degradation of IκB-α or both. IκB-α degradation exposes a nuclear localization signal and leads to the activation of NF-κB. Thus, we also investigated whether rapamycin modulates NF-κB activity in BV2 cells by inhibiting the degradation of IκB-α [25]. As expected, rapamycin significantly inhibited the degradation of IκB-α in BV2 cells. These results indicate that rapamycin is capable of inhibiting microglial activation by modulating NF-κB signaling.

### The Inhibition of Microglial Activation by Rapamycin is Involved in Rapamycin–mediated Neuroprotection in the COH

**Figure 4:**
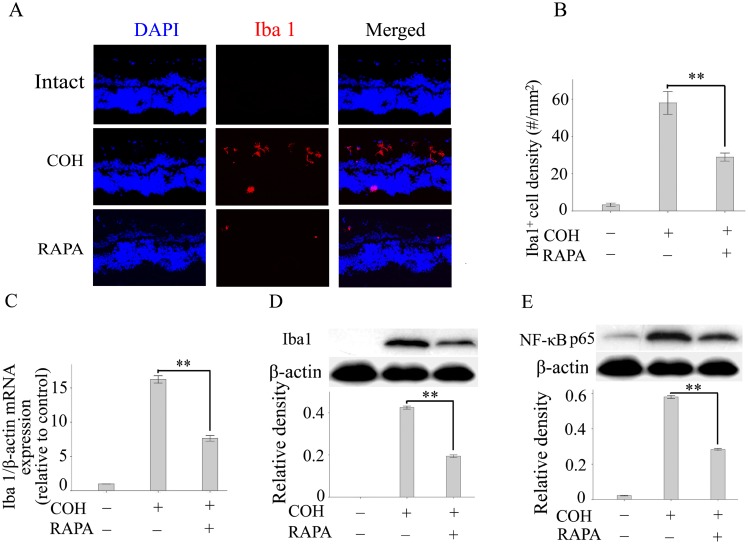
Inhibition of microglial activation by rapamycin is involved in rapamycin–mediated neuroprotection in the COH. (A): Representative images of Iba1 immunostaining (red) of retinas in a normal rat, a COH rat, and a COH rat after treatment with rapamycin. (B): Quantification of Iba1^+^ cells in the retinas of different experimental groups. The expression of Iba1 mRNA (C) and protein (D) in the retinas of different experimental groups was determined by real-time PCR and western blotting, respectively. (E): The expression of NF-κB in retinas was determined by western blotting. Each group includes 8 rats, and experiments were repeated once. Values are presented as the mean ± SEM of 3 replicates. **p<0.01. Abbreviations: COH, chronic ocular hypertension model; RAPA, rapamycin; NF-κB, nuclear factor-kappa B; Iba1, ionized calcium-binding adapter molecule 1.

Next, we further explored whether inhibition of microglial activation is involved in rapamycin–mediated neuroprotection in experimental glaucoma. Iba1, which is expressed and upregulated in activated microglia, has been considered a specific marker for activated microglia [26]–[28]. Thus, to monitor microglial activation in experimental glaucoma, we quantified the levels of Iba1 mRNA and protein in retinas after 6 weeks of rapamycin or vehicle treatment. The results from real-time PCR showed a significant increase in Iba1 expression in retinas in glaucoma, whereas rapamycin suppressed upregulation of Iba1 expression. Immunostaining showed that rapamycin treatment robustly reduced the number of Iba1+ microglia compared with the untreated group. Western blotting results also further confirmed a rapamycin-mediated inhibitory effect on microglial activation. Because our *in* *vitro* studies have shown that rapamycin suppresses microglial activation by inhibiting NF-κB signaling, we next investigated whether this mechanism is also implicated in rapamycin–mediated neuroprotective effects in experimental glaucoma. As shown in Figure 4E, treatment with rapamycin significantly inhibited NF-κB activation. These results suggest that inhibition of microglial activation is involved in rapamycin–mediated neuroprotection in experimental glaucoma.

### Rapamycin Inhibited Apoptosis in Glutamate-injured Primary RGCs

**Figure 5:**
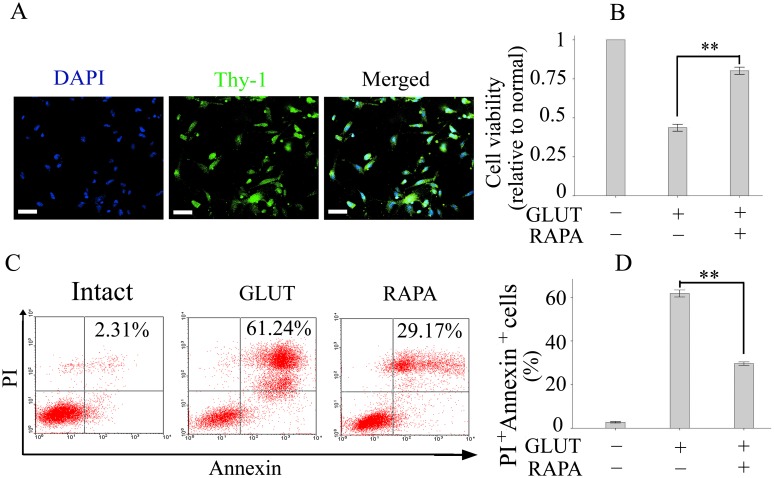
Rapamycin inhibited apoptosis in glutamate-injured primary RGCs. (A): Representative images of Thy-1 immunostaining (green) in primary RGCs. (B): The cell viability of primary RGCs was measured by MTT assay. (C, D): The numbers of PI^+^Annexin^+^ cells, indicating apoptosis, were detected by flow cytometry. Values are presented as the mean ± SEM of 3 replicates, and experiments were repeated at least 2 times with similar results. **p<0.01. Abbreviations: GLUT, glutamate; RAPA, rapamycin; PI, propidium iodide.

Next, we performed a series of *in* *vitro* studies to evaluate whether rapamycin is capable of directly inhibiting the apoptosis of RGCs and to explore the underlying mechanisms. Primary RGCs were cultured and used for this purpose. We determined whether primary RGC cells express characteristic markers of retinal ganglion cells by immunostaining with Thy-1 antibodies. Immunostaining results indicated that primary RGCs indeed expressed Thy-1 (Fig. 5A).

We next exposed primary RGCs to glutamate (100 µM) for 24 h to induce apoptosis. As expected, glutamate decreased the cell viability and rapamycin (100 nM) treatment significantly increased the cell viability in a dose-dependent manner as measured by MTT assay (Fig. 5B). The results from flow cytometry also showed that the numbers of PI^+^Annexin^+^ cells, indicating apoptosis, were increased in primary RGCs after glutamate exposure (Fig. 5C, 5D). Treatment with rapamycin significantly decreased the numbers of PI^+^Annexin^+^ cells as assayed by flow cytometry (Fig. 5C, 5D).

### The Inhibition of Akt Dephosphorylation is Implicated in Rapamycin-mediated Neuroprotection in Primary RGCs and the COH

**Figure 6:**
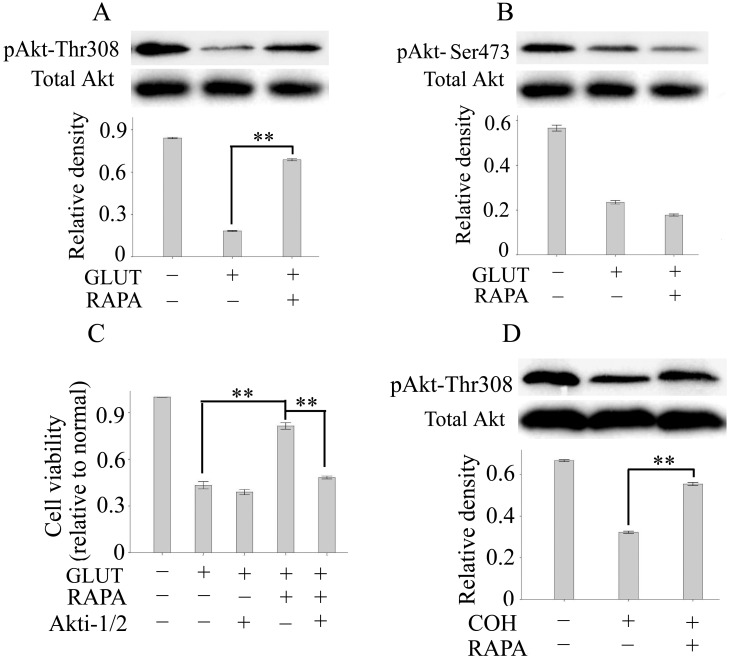
Inhibition of Akt dephosphorylation is implicated in rapamycin-mediated neuroprotection in primary RGCs and the COH. Akt phosphorylation at sites Thr308 (A) and Ser473 (B) in primary RGCs was determined by western blotting. (C): The cell viability of primary RGCs was measured by MTT assay. (D): Akt phosphorylation at Thr308 in retinas was measured by western blotting. Values are presented as the mean ± SEM of 3 replicates, and experiments were repeated at least 2 times with similar results. **p<0.01. Abbreviations: GLUT, glutamate; RAPA, rapamycin; Akti-1/2, an Akt inhibitor.

Previous studies haves shown that the pro-survival kinase Akt is critical in neuron survival and death. We therefore examined Akt phosphorylation in primary RGCs exposed to glutamate and rapamycin. The results from western blotting showed that glutamate repressed Akt phosphorylation at both Thr308 and Ser473. Treatment with rapamycin (100 nM) significantly prevented glutamate-mediated dephosphorylation of Akt at Thr308 but not Ser473 (Fig. 6A, 6B). These findings indicate that rapamycin may be neuroprotective because it maintains Akt phosphorylation at Thr308 (the site regulated by growth factors through PI3k signaling), which is sufficient to activate the kinase and to maintain cell survival [29]. To further confirm the role of Akt phosphorylation maintenance in rapamycin-mediated neuroprotection, an Akt inhibitor (Akti-1/2) was used. Our results showed that Akti-1/2 significantly reversed rapamycin-mediated neuroprotection in primary RGCs after glutamate exposure (Fig. 6C). We next investigated whether modulation of Akt is also implicated in rapamycin–mediated neuroprotective effects in experimental glaucoma. We quantified the levels of Thr308-phosphorylated Akt in retinas after 6 weeks of rapamycin or vehicle treatment. Western blotting results showed a significant decrease in phosphorylated Akt expression in retinas in glaucoma, whereas rapamycin upregulated phosphorylated Akt expression. These results implicate Akt phosphorylation maintenance at Thr308 in rapamycin-mediated neuroprotection in primary RGCs and experimental glaucoma.

## Discussion and Conclusions

The progressive loss of RGCs is responsible for visual field loss and irreversible blindness in glaucoma. The developments of new strategies that prevent or delay RGC death have been recognized as an important approach to glaucoma therapy [1], [2]. Rapamycin, an FDA-approved drug, is a macrolide antibiotic that was first developed as an antifungal agent. However, decades of accumulating evidence have shown that rapamycin possesses versatile nonantibiotic properties, including immunosuppression [30], anti-proliferation [31] and extension of lifespan [32]. Recently, rapamycin-mediated neuroprotective effects in the CNS and peripheral nervous system have received increasing attention [5], [8]. However, the neuroprotective effects of rapamycin in glaucoma and their underlying mechanisms remain elusive. Here, we demonstrated that in experimental glaucoma, treatment with rapamycin significantly reduced RGC loss, down-regulated active caspase-3 expression, and inhibited the production of neurotoxic mediators, including TNF-α, NO and ROS. The results of *in* *vitro* studies of primary RGCs showed that rapamycin was capable of directly suppressing the apoptosis of primary RGCs induced by glutamate. To our knowledge, these findings provide reliable evidence that rapamycin is neuroprotective in experimental glaucoma.

Microglia, the immune surveillance cells of the CNS and retina, are normally quiescent but become readily activated during most neuropathologic conditions, including peripheral nerve injury, trauma, inflammatory disease, and neurotoxicant-induced neuronal injury [33], [34]. During activation, microglia exhibit changes in cell morphology, cell surface receptor expression, and the production of mediators. In glaucoma, microglial activation and microglia-derived neurotoxic mediators, including TNF-α, NO and ROS, have been shown to play detrimental roles [35]–[37]. Previous studies have shown that inhibition of microglial activation protects RGCs from death in several models of glaucoma [18]–[20]. In this study, we demonstrated that rapamycin significantly inhibited TNF-α and NO release in microglia activated by LPS *in* *vitro*. Mechanistically, we found that rapamycin may inhibit microglial activation by modulating NF-κB signaling. Importantly, we also observed that rapamycin treatment led to significant suppression in microglial activation in the COH, which was manifested as a significant decrease in the expression of Iba1 (a specific marker for activated microglia) and NF-κB activation. These findings indicated that inhibition of microglial activation by rapamycin is involved in rapamycin–mediated neuroprotection in experimental glaucoma.

The PI3k/Akt pathway is an important signal transduction pathway that regulates cell survival of neurons and of other cell types [38], [39]. The phosphorylation of Akt is a critical step in the PI3k/Akt pathway for maintaining cell survival. Accumulating evidence has shown that the phosphorylation of Akt is gradually decreased in several chronic neurodegenerative diseases, including Alzheimer’s disease, Parkinson’s disease and glaucoma [40], [41]. Thus, increase or maintenance of Akt phosphorylation has been regarded as an important neuroprotective target in these diseases. Akt has two phosphorylation sites, Ser473 and Thr308. Generally, the phosphorylation of Akt at both Ser473 and Thr308 provides maximum catalytic activity. However, Akt phosphorylation at the Thr308 site, a target for the phosphatidylinositol 3-kinase (PI3K)–PDK1 pathway [42], is sufficient to promote Akt activity and cell survival [29]. In this study, we showed that glutamate, the major excitatory neurotransmitter in glaucoma, repressed Akt phosphorylation at both Ser473 and Thr308, which is consistent with the findings of previous studies. Treatment with rapamycin prevented glutamate-mediated dephosphorylation at Thr308 but not Ser473. Furthermore, rapamycin-mediated neuroprotection in primary RGCs was reversed by an Akt inhibitor (Akti-1/2). Meanwhile, rapamycin also significantly upregulated phosphorylated Akt expression at Thr308 in the COH. These results suggested that maintaining Akt phosphorylation at Thr308 might contribute, at least in part, to the underlying mechanism of rapamycin-mediated neuroprotection in primary RGCs and the COH.

Autophagy, a regulated process for degradation and recycling of cellular constituents, is the major intracellular degradation system. Previous studies show that autophagy plays important role in neurodegeneration diseases. Rapamycin, used as an autophagy enhancer, have been shown to play neuroprotective roles in neurogeneration diseases [43]–[46], including laser-induced glaucoma [47] and optic nerve axotomy [48]. In addition, Han et al. reported that enhancing autophagy may be involved in rapamycin-mediated inhibition of BV2 microglia activation [49]. Our group recently reported that autophagy is significantly enhanced in a Rhesus monkey chronic hypertensive glaucoma model [50]. In this study, we also found that rapamycin significantly enhanced autophagy in a rat chronic hypertensive glaucoma model (data not shown). Take together, this suggest that enhancing autophagy may be a mechanism of rapamycin-mediated neuroprotection in experimental glaucoma. However, the roles of autophagy in glaucoma remain controversial. Several studies show that autophagy contributes to RGC death of glaucoma, and inhition of autophagy decreases RGC apoptosis [51]–[53]. Importantly, using the same model as our study, Park et al. reported that inhibition of autophagy is neuroprotective in glaucoma [54]. In addition, rapamycin possesses multiple biological functions and enhancing autophagy may be only one of the important biological properties of rapamycin. Therefore, one should be careful when interpreting the role of autophagy in rapamycin-mediated neuroprotection in glaucoma.

According to previous studies, the results of FG-labeled RGC is a direct readout of axonal damage and can also grossly estimate RGC number in optic nerve injuries. Thus, the increased RGC density by rapamycin reveals directly an improvement in axonal transport in COH. In addition, it is also possible that rapamycin protects both axons and somas of RGC in COH. Further studies are warranted to dissect the detailed mechanisms.

In conclusion, this study provides the compelling evidence that rapamycin is neuroprotective in experimental glaucoma. This protective action appears to be attributable to direct suppression of RGC apoptosis and the production of neurotoxic mediators by microglia. These findings further elucidate the neuroprotective mechanism of rapamycin and support the notion that rapamycin is potentially therapeutic for patients with glaucoma.
