# Correction: Rapamycin Is Neuroprotective in a Rat Chronic Hypertensive Glaucoma Model

**DOI:** 10.1371/journal.pone.0213489

**Published:** 2019-03-04

**Authors:** Wenru Su, Zuohong Li, Yu Jia, Yehong Zhuo

There is an error in Fig 4A of this article [1]. The top row of panels are incorrectly labelled “Intact”. These images were taken from an adjacent section of the same sample as the "COH" images, but are stained with isotype control antibody. A corrected Fig 4 is provided in which these images are re-labelled as isotype control. Images for a stained section from intact rat retina collected in the same experiment are now added.

The authors provide the following methodological clarification: Some Western blot β-actin loading control images are duplicated within the figures (see Figs 3D–3F, 4D, 4E, 6A and 6B) because target proteins were probed on the same gels, and therefore have the same β-actin loading controls.

The underlying data files for the article are provided as Supporting Information.

A member of PLOS ONE’s Editorial Board confirms that the revised figures and the raw data support the results and conclusions of the published article.

**Fig 4:**
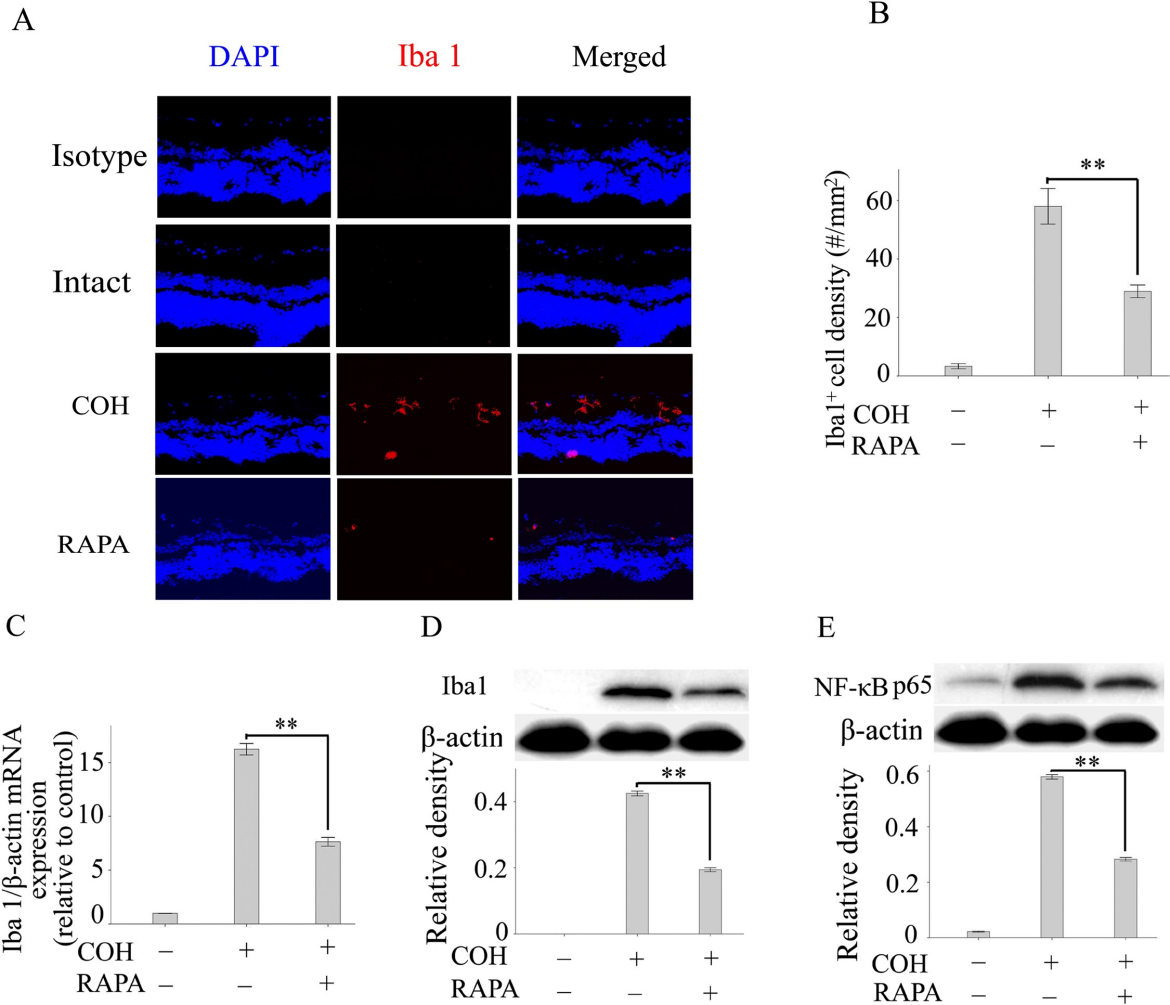
Inhibition of microglial activation by rapamycin is involved in rapamycin–mediated neuroprotection in the COH. (A): Representative images of Iba1 immunostaining (red) of retinas in isotype control, an intact rat, a COH at, and a COH rat after treatment with rapamycin. (B): Quantification of Iba1+ cells in the retinas of different experimental groups. The expression of Iba1 mRNA (C) and protein (D) in the retinas of different experimental groups was determined by real-time PCR and western blotting, respectively. (E): The expression of NF-κB in retinas was determined by western blotting. Each group includes 8 rats, and experiments were repeated once. Values are presented as the mean ± SEM of 3 replicates. **p<0.01. Abbreviations: COH, chronic ocular hypertension model; RAPA, rapamycin; NF-κB, nuclear factor-kappa B; Iba1, ionized calcium-binding adapter molecule 1.

## Supporting information

S1 File Dataset. Raw data for Figs 1–6.
(RAR)

S2 File Dataset. Raw data for Fig 4A.
(RAR)

S3 File Dataset. Further raw data for Fig 4A.
(RAR)
